# Protein Ubiquitylation in Pancreatic Cancer

**DOI:** 10.1100/tsw.2010.133

**Published:** 2010-07-20

**Authors:** Thomas Bonacci, Julie Roignot, Philippe Soubeyran

**Affiliations:** INSERM U624, Cellular Stress, Parc Scientifique et Technologique de Luminy, Marseille, France

**Keywords:** cancer, pancreas, ubiquitin, ubiquitylation

## Abstract

Pancreatic cancer is one of the worst, as almost 100% of patients will die within 5 years after diagnosis. The tumors are characterized by an early, invasive, and metastatic phenotype, and extreme resistance to all known anticancer therapies. Therefore, there is an urgent need to develop new investigative strategies in order to identify new molecular targets and, possibly, new drugs to fight this disease efficiently. Whereas it has been known for more than 3 decades now, ubiquitylation is a post-translational modification of protein that only recently emerged as a major regulator of many biological functions, dependent and independent on the proteasome, whose failure is involved in many human diseases, including cancer. Indeed, despite its role in promoting protein degradation through the proteasome, ubiquitylation is now known to regulate diverse cellular processes, such as membrane protein endocytosis and intracellular trafficking, assembly of protein complexes, gene transcription, and activation or inactivation of enzymes. Taking into account that ubiquitylation machinery is a three-step process involving hundreds of proteins, which is countered by numerous ubiquitin hydrolases, and that the function of ubiquitylation relies on the recognition of the ubiquitin signals by hundreds of proteins containing a ubiquitin binding domain (including the proteasome), the number of possible therapeutic targets is exceptionally vast and will need to be explored carefully for each disease. In the case of pancreatic cancer, the study and the identification of specific alteration(s) in protein ubiquitylation may help to explain its severity and may furnish more specific targets for more efficient therapies.

## INTRODUCTION

Pancreatic cancer, also called PDAC (for pancreatic ductal adenocarcinoma), belongs to the worst forms of cancer[1], with a 6- to 8-month life expectancy from diagnosis and less than 5% of 5-year survival. Among the poor-prognosis cancers, pancreatic cancer has the highest incidence in the population of western countries. At present, this cancer is at the tenth position in terms of new cases, but, strikingly, at the fourth position in terms of death by cancer[1]. When discovered, pancreatic tumors are often already invasive and metastatic, and are highly resistant to conventional therapy[2]. Currently, only gemcitabine, an analogue of deoxycytidine (2', 2'-difluoro-2'-deoxycytidine), has been demonstrated to improve the life expectancy of patients slightly[3]. Finding a way to cure or, at least, to prolong the survival of pancreatic cancer patients is still challenging the scientific and medical communities.

As for other cancer types, genetic and genomic analyses of pancreatic cancer have revealed a set of altered genes, by mutations or changes in expression[4,5]. They include the activation of oncogenes, such as K-Ras (which is constitutively activated in about 95% of tumor samples), and the inhibition of tumor suppressors, such as INK4A (P16) (which is inactivated in almost all pancreatic tumors) and P53 or DPC4 (SMAD4) (which are inactivated in more than 60% of cases)[6,7,8]. However, the acquired knowledge and the development of new drugs have not improved, so far, the treatment and the outcome of this disease. All these failures are clues that have driven scientists to think of a particular nature of pancreatic cancer[2] that would explain why it is so rapidly invasive, metastatic, and resistant. It is becoming obvious that new approaches are needed to decipher the particular mechanisms responsible for these unique characteristics of pancreatic cancer.

Among the emerging biological fields of investigation that popped out these last years (hypoxia, autophagy, microenvironment, etc.), some, if not all, should be applicable to pancreatic cancer research. Post-translational modifications (PTMs) of proteins, such as phosphorylation or acetylation, are central biological regulatory processes[9] and failures in these processes have been extensively described for oncogenic diseases. One of the most documented of the PTMs is definitely the phosphorylation of proteins on serine/threonine/tyrosine residues, and deregulation of this process has been described for many pathological situations, mainly in human cancer[10].

Protein ubiquitylation is one of these PTMs, discovered in the early 1970s and long since known to be part of the ubiquitin-proteasome system, involved in the turnover of many proteins. However, the past decade has revealed that what was known about the ubiquitylation system was just the tip of the iceberg. As described below, protein ubiquitylation regulates a tremendous number of biological processes in a proteasome-dependent, but also -independent manner. Indeed, beyond the mere induction of protein degradation by the proteasome, ubiquitylation of proteins is involved in regulating many biological processes, such as activity, specificity, localization, and interactions of target proteins[11]. Defects of the ubiquitylation system were and are found in a growing number of well-known diseases[12]. Therefore, it is highly probable that defects in protein ubiquitylation can actively participate in the extreme aggressiveness of pancreatic cancer cells, and to exploit this new field of investigation should be of primary importance.

## UBIQUITIN AND UBIQUITIN-LIKE PROTEINS

Ubiquitin is a 76-amino-acid protein with a molecular weight of 8.5 kDa, first isolated at the beginning of the 1970s from bovine thymus and was termed thymin[13]. Shortly after, its ubiquitous nature, present in all cells of all eukaryotes, led to its definitive name, ubiquitin, and the ATP-dependant proteolytic system dependent on ubiquitin was termed the ubiquitin proteasome system (UPS)[14].

Ubiquitylation is the formation of an isopeptide bond between the COOH group of the C-terminal glycine of ubiquitin and the NH2 group of a lysine residue of the target protein. Several types of ubiquitylation may modify target proteins. They include monoubiquitylation, multiple monoubiquitylation, or polyubiquitylation (chains of ubiquitin) that can be of various length and different types (depending on the lysine residue of ubiquitin, out of seven, which is engaged in the binding with the next ubiquitin)[15].

Monoubiquitylation is mainly involved in the regulation of endocytosis, lysosomal targeting, meiosis, and chromatin remodeling, whereas polyubiquitylation is implicated in targeting modified proteins to proteasomal degradation, immune signaling, and DNA repair (Fig. 1).

Ubiquitylation involves the successive activity of three enzymes: an ubiquitin-activating enzyme, also named “E1” (two in the human genome), a conjugating enzyme “E2” (at least 38), and an ubiquitin ligase “E3” (600–1000)[16], whose action is balanced by ubiquitin hydrolases, also named deubiquitylating enzymes (DUBs)[17]. Two major types of ubiquitin ligases have been described: HECT domain type and the RING domain type[15]. DUBs are divided into five subclasses: the ubiquitin C-terminal hydrolases (UCHs), the ubiquitin-specific proteases (USPs), the Machado-Joseph Disease protein domain proteases (MJDs), the ovarian tumor proteases (OTUs), and the JAMM motif proteases[17].

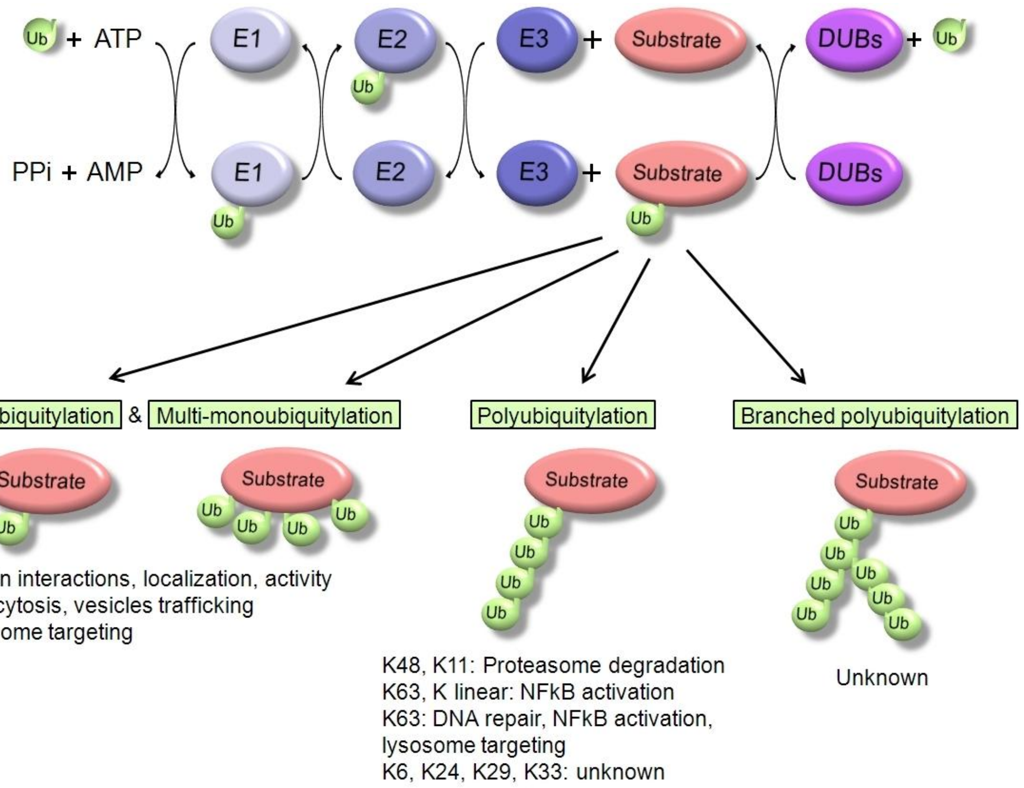

**FIGURE 1.** The ubiquitylation machinery and associated functions. Before being conjugated to the target protein, ubiquitin needs to go through a three-step process that implicates first the attachment of ubiquitin to a ubiquitin-activating enzyme (E1) via the hydrolysis of one ATP molecule. Subsequently, the ubiquitin is transferred to a conjugating enzyme (E2) and then the ubiquitin ligase (E3) will enable the transfer of ubiquitin to a specific target protein. This process, which leads to monoubiquitylation of the substrate, may be repeated to produce either multimonoubiquitylation or polyubiquitylation of the same protein. This protein modification can be removed by the action of DUBs. These different forms of ubiquitylation, depending on the nature of the target protein, will have miscellaneous functions.

Ubiquitin-like protein modifiers (ULMs) are closer to ubiquitin in terms of ternary structure, the so-called ubiquitin fold, than in terms of primary sequence[18]. They also share the C-terminal glycine whose carboxyl group forms the isopeptide bond with the ε-amino group of the lysine residue of the modified protein. Modifications by ULMs follow a similar scheme to that of ubiquitylation (activation-conjugation-ligation balanced by hydrolases), using either specific and/or common enzymes. Ligation of ubiquitin-like proteins on target proteins can have as many consequences as with ubiquitylation, protein interaction, activity, localization, and degradation. ISG15 (a diubiquitin molecule) was the first ULM identified in the late 1980s as an interferon-stimulated protein of 15 kDa[19]. Among the nine families of ubiquitin-like molecules[18], the most renowned is the SUMO (small ubiquitin-like modifier 1) family, which contains four members: SUMO-1 (the most studied), SUMO-2, SUMO-3, and SUMO-4, whose polymorphism maybe be correlated to susceptibility to type I diabetes[20].

Finally, the variable functions provided by the various types of ubiquitin and ubiquitin-like modifications are principally based on their specific recognition by a growing number of ubiquitin/ubiquitin-like binding domains (UBD) contained in many proteins[21]. These UBDs may preferentially bind to monoubiquitin, multimonoubiquitin, or specific polyubiquitin chains, thereby mediating the versatile functions of ubiquitin in the cell.

## UBIQUITIN IN PATHOLOGICAL SITUATIONS

Ubiquitylation was first described as a process targeting proteins for degradation through the proteasome. However, in recent years, ubiquitylation has been implicated in the regulation of other biological functions, such as intracellular trafficking, assembly of signaling protein complexes, and activation or inactivation of enzymes. This may explain why ubiquitin has also been involved in many different human diseases.

Deficiencies in the UPS have been largely described in different neurodegenerative diseases, such as Alzheimer's, Parkinson's, or Huntington's diseases. Accumulation of ubiquitylated TAU protein and aggregation of amyloid-β protein generate neurofibrillary plaques that protect these proteins from proteasomal degradation; this abnormal accumulation of TAU protein is responsible for Alzheimer's disease[22]. In juvenile Parkinson's disease, mutations of the ubiquitin ligase parkin are responsible for its inactivation, suggesting that its substrates might no longer be ubiquitylated and degraded. These proteins could then accumulate, in a fashion similar to that in Alzheimer's disease, and induce cellular toxicity and neuronal loss[23]. Finally, in Huntington's disease, the expansion of polyglutamine repeats in the huntingtin protein causes the formation of protein aggregates, named inclusion bodies, that are resistant to proteasomal degradation, impairing its function[24].

Ubiquitylation is involved in mechanisms that control the physiological balance of the body. By controlling the stability of IRS (insulin receptor substrate) proteins, the UPS has been shown to play a key role in the regulation of insulin signaling and insulin secretion by the pancreatic β-cells, and, therefore, in the regulation of body glycemia[25]. Deregulation of the UPS is also involved in the decrease of skeletal muscle associated with cancer (discussed below) or aging[26]. Cystic fibrosis, a common hereditary disease that affects the entire body, is caused by mutations in the CFTR receptor (cystic fibrosis transmembrane receptor), a chloride ion channel. These mutations generate misfolded proteins that are degraded via the quality-control pathway of the UPS[27]. Inversely, Liddle's syndrome is due to a mutation in a sodium ion channel (ENaC) that avoids its recognition by the Nedd4 ubiquitin ligase and leads to its abnormal accumulation at the plasma membrane[28]. This generates an increased sodium reabsorption by the kidneys, which leads to hypertension due to an increase in extracellular volume.

As a major modulator of the NF-κB (nuclear factor-kappaB) pathway, ubiquitin plays an important role in the regulation of inflammation and innate immunity. Indeed, ubiquitylation initiates the proteasomal degradation of the inhibitor of NF-κB, IκBα[29], or enables the processing of NF-κB precursors[30]. Additionally, ubiquitylation can also regulate the NF-κB signaling pathway in a proteasome-independent manner[31].

The ubiquitin system is one of the cellular mechanisms that viruses may hijack for their own benefit and to propagate themselves. Two HIV proteins, VIF and VPU, were shown to use different cullin-RING ligases to mediate the ubiquitylation of cellular proteins. VIF facilitates the ubiquitin-dependent proteasome degradation of the antiretroviral enzyme APOBEC3G by interacting with Cul5-ElonginB and C[32]. VPU forms a trimeric complex with the CD4 coreceptor molecule for HIV and the F-box protein β-TRCP to down-regulate the cell surface expression of CD4, hence enhancing the evasion of newly formed viral particles[33,34]. Viruses can also synthesize their own ubiquitin ligases, such as the Kaposi's sarcoma herpesvirus, which encodes two ubiquitin ligases, K3 (MIR1) and K5 (MIR5), that mediate the down-regulation of several immunoreceptors (MHC classI, ICAM1) from infected cells[35,36].

Like protein phosphorylation, protein ubiquitylation is largely involved in many pathways that regulate apoptosis and cell cycle, and, as such, alterations of protein ubiquitylation are observed in most, if not all, types of cancers, including pancreatic cancers[37]. One of the best examples is represented by the P53-MDM2 couple. P53 is a well-known tumor suppressor gene mutated in more than 50% of tumors, whereas MDM2 is a known oncogene that acts as one of the ubiquitin ligases that mediate the ubiquitylation of P53 responsible for its degradation by the proteasome[38,39]. Accordingly, high MDM2 levels are observed in many tumors. The UPS plays a central role in controlling the cell cycle. Two ubiquitin ligase complexes, named SCF (SKP1 CUL1 F-box) and APC/C (anaphase promoting complex/cylcosome), execute a timely controlled program that leads to the successive degradation of key cell cycle proteins[40]. The functions of the different SCF complexes depend on their F-box protein, and the SKP2-containing SCF complex is the most determinant for cell cycle as its major ubiquitylation substrate is the Cdk inhibitor p27. Only two forms of APC/C exist, APC/$C^{Cdc20}$ and APC/$C^{Cdh1}$, playing distinct roles during the cell cycle. Importantly, different members from both complexes were found to be mutated in cancer[41,42,43].

## UBIQUITIN AND PANCREATIC CANCER CACHEXIA

At the physiological level, the UPS has been described in pancreatic cancer as a major mediator of skeletal protein degradation in cachexia. Cachexia is a syndrome characterized by a progressive loss of adipose tissue and skeletal muscle, leading to a general reduction of body weight[44]. This syndrome, usually associated with anorexia, strongly reduces the quality of life and survival of patients. Pancreatic cancer has the highest incidence of cachexia[45], with 80% of cachectic patients at the time of diagnosis. The loss of skeletal muscle is responsible for the lower mobility of patients (explaining the poor quality of life) and the loss of respiratory function that leads to death by respiratory failure in 48% of cancer patients[46].

Different factors secreted by the tumor were shown to possibly induce muscle atrophy, such as TNF-α, proteolysis-inducing factor (PIF), IL-6, angiotensin II, or glucocorticoids. PIF is a 24-kDa sulfated glycoprotein originally isolated from a cachexia-inducing MAC16 tumor[47]. It was present in the urine of cancer patients with cachexia, but absent from the urine of patients with the same tumor type, but without cachexia[48]. PIF was shown to play a pivotal role in protein catabolism in cancer cachexia by inducing the ubiquitin-proteasome pathway in muscle cells[49]. Two major ubiquitin ligases, muscle atrophy F box (MAFbx)[50] and muscle RING finger 1 (MuRF1)[51], were shown to be highly expressed during muscle atrophy. Their implication in this process has been confirmed by the observation that mice deficient for one of these two genes are resistant to atrophy[50]. Myosin heavy chain is a validated target for MuRF1[52], which is also able to ubiquitinylate troponin 1[53]. Finally, a TNF-α and IL-6 inducible ubiquitin ligase, E3α-II, also has an increased level in muscles during cancer cachexia[54].

## UBIQUITIN-DEPENDENT SIGNALING IN PANCREATIC TUMORS

Among the signaling pathways that are altered in pancreatic tumors, many involve aberrant ubiquitylation of signaling molecules. SMAD4, a major mediator of TGF-β signaling involved in the regulation of cell proliferation, is well known to be mutated or deleted in about 50% of pancreatic cancers[55]. A missense mutation in a conserved arginine residue of SMAD4 increases its polyubiquitylation by the ligase UbcH5, leading to its accelerated proteasomal degradation[56]. Moreover, a nonsense mutation in SMAD4 was shown to generate a truncated form that is rapidly degraded through the UPS[57]. SCF (β-TRCP1) is another ubiquitin ligase that is critical for the degradation of SMAD4 protein in pancreatic cancer cells. Its interaction with SMAD4 is mediated by the F-box protein β-TRCP1, contained in this SCF complex. Most point mutations in SMAD4 increase the affinity for β-TRCP1, resulting in an important ubiquitylation by SCF and in the inhibition of TGF-β biological activity in pancreatic cancer cells[58]. β-TRCP1 has also been associated with the permanent activation of NF-κB and could account for the intense resistance of pancreatic cancer cells towards chemotherapy[59]. Chemoresistant pancreatic cancer cell lines exhibiting constitutive NF-κB activity were shown to express significantly elevated levels of β-TRCP1 when compared with chemosensitive pancreatic carcinoma cell lines lacking constitutive NF-κB activity[60].

Oncogenic transformation is usually accompanied by loss of cell polarity. Gelsolin is an actin filament–capping protein that plays an important role in maintaining cytoskeletal integrity necessary for sustained cell polarity. The high ubiquitin-proteasome activity observed in pancreatic tumors or cancer cell lines was shown to be responsible for the decrease in gelsolin protein level and loss of cytoskeletal integrity[61].

A great majority of pancreatic tumors and tumor cell lines express elevated levels of transglutaminase (TG2). This TG2 overexpression promotes the degradation of the phosphatase PTEN by the ubiquitin-proteasome pathway, which results in constitutive activation of focal adhesion kinase/AKT cell survival signaling[62].

Siah2 is a RING-finger ligase, which is an important regulator of pathways activated under hypoxia. It regulates PHD3 and PHD1 (prolyl hydroxylases) under low oxygen concentration, thereby allowing accumulation of hypoxia-inducible factor (HIF)-1α, which regulates the hypoxia response within the range of physiological normoxic to mild hypoxic conditions[63]. Considering that pancreatic tumors are some of the less vascularized tumors and therefore the most hypoxic[64], it is not surprising that such a ligase plays an important role in the development of pancreatic cancer[63]. Inhibition of Siah-dependent proteolysis abolished the tumor growth of human pancreatic cancer cells in soft agar as well as in xenografted NUDE mice[65]. Siah1 interacting protein (SIP) was also found to be highly expressed in several tumoral tissues, including pancreatic cancer[66]. SIP acts as an adaptor between Siah and B-catenin, thereby facilitating its ubiquitylation-dependant proteasomal degradation[67].

Pim-1 is a proto-oncogene that regulates apoptosis and the cell cycle by phosphorylating target proteins, and overexpression of Pim-1 can cause genomic instability. Hypoxia was shown to stabilize Pim-1 by preventing its ubiquitin-mediated proteasomal degradation. Blocking Pim-1 function by introduction of dominant-negative Pim-1 resensitized pancreatic cancer cells to apoptosis induced by glucose deprivation under hypoxia and reduced their tumorigenicity *in vivo*[68].

Recent findings showed that RNF13, a RING-type ubiquitin ligase, is overexpressed in a significant number of pancreatic tumors and displays a good correlation with tumor grade[69]. Authors could demonstrate that this ligase may possibly play an important role for pancreatic progression as overexpression of the wild-type protein, but not the inactive RING-deleted mutant, could promote the invasive potential of a pancreatic cancer cell line by increasing the activity of metalloproteinase-9[69].

The identification of all these punctual alterations of the ubiquitin system represents new potential targets for the development of more specific therapies. Moreover, it is likely that future research will have an impact on the identification of new failures in the ubiquitin system.

## PROTEASOME INHIBITORS AND PANCREATIC CANCER

Treatment with proteasome inhibitors results in stabilization and accumulation of proteasome substrates, a phenomenon that may result in cell cycle arrest and activation of apoptotic programs[70,71]. Bortezomib (VELCADE, PS-341) is a small, cell-permeable molecule that specifically and selectively inhibits the proteasome by binding in a reversible manner. Initial screening of bortezomib against a panel of tumor cells demonstrated its activity against a broad range of tumor types[72]. Subsequent studies have shown that bortezomib was efficient in various *in vitro* and *in vivo* tumor models, including pancreatic cancer[73]. Based on these findings, preclinical studies were performed and showed that use of bortezomib in pancreatic cancer could induce apoptosis and enhance sensitivity to chemotherapy[74,75]. Whereas bortezomib could strongly reduce the development of BxPC3 pancreatic cancer cells xenografted in a mouse by itself[74], it only slightly increased the response to gemcitabine[75] in a mouse xenograft model that used MIAPaCa-2 pancreatic cancer cells.

Proteasome inhibition was first shown to enhance TNF-α–induced apoptosis in a pancreatic cancer cell line by inhibiting the degradation of the ubiquitylated IκB (inhibitor of NF-κB), thus blocking the desinhibition of NF-κB[76]. The inhibition of NF-κB activation was found to be a crucial mechanism in inducing apoptosis and in overcoming resistance mechanisms in multiple myeloma[77].

Despite these encouraging findings, contrasting results came from a phase II trial (of the North Central Cancer Treatment Group) that investigated bortezomib monotherapy vs. bortezomib + gemcitabine in patients with metastatic pancreatic carcinoma. Patients who received bortezomib monotherapy had a median survival of 2.4 months, while the survival was 4.8 months when gemcitabine was added[78]. These results are specially disappointing when compared to the usual 6-month survival with gemcitabine treatment alone[3].

Soon after this first drawback, it was shown that pancreatic cancer cells exposed to bortezomib formed aggregates of ubiquitin-conjugated proteins (aggresomes) *in vitro* and *in vivo*, which played a cytoprotective role[79]. As these aggresomes could be disrupted by gene silencing of histone deacetylase 6 (HDAC) or by chemical HDAC inhibitors, which resulted in increased efficacy of bortezomib against pancreatic cancer cells, it has been suggested that treatment with bortezomib in combination with HDAC inhibitors could be a way to circumvent the protumoral effect of bortezomib alone[79].

In accordance with this complete failure of bortezomib in this phase II trial, it was recently shown that whereas bortezomib inhibits growth and induces apoptosis in pancreatic cancer cells *in vitro*, it displayed a significant tumor-promoting effect in an orthotopic pancreatic cancer mouse model[80]. This unexpected deleterious effect of bortezomib was associated with the induction of angiogenesis and expression of angiogenic factors. Elevated microvessel density is known to correlate with a poor prognosis in patients with pancreatic cancer[81]. These results contrast with the effect of bortezomib on multiple myeloma cells where it was shown to inhibit angiogenesis[82].

Moreover, besides the promotion of angiogenesis, it has been demonstrated that inhibition of the proteasome by either bortezomib or NPI-0052 (a second-generation inhibitor) in pancreatic cancer cells leads to the activation of antiapoptotic and mitogenic signaling pathways[83]. These include the activation of the EGF receptor, the ERK MAP kinases, and the PI3 kinase. The activation of these pathways actively contributes to the inefficiency of these proteasome inhibitor treatments[83].

Therefore, despite the success of inhibiting the complete proteasome for treating certain types of cancers, such as multiple myeloma[84], the use of such a strategy for pancreatic cancer seems definitely inappropriate. Fortunately, new proteasome inhibitors that would be more efficient against cancer cells and less toxic for normal tissues have been and will be discovered. One example is the new promising proteasome inhibitor PI-083, which appeared to inhibit specifically the chymotrypsin-like activity in transformed cells over nontransformed immortalized cells[85].

## PERSPECTIVES

The most promising aspect regarding the discovery of new inhibitors relies on specific molecular targets of the ubiquitin system, independently of the proteasome. Indeed, as a multistep process, involving several E1s, more E2s, and much more E3s, as well as DUBs and a plethora of substrates, many of them being potentially altered in cancer, the ubiquitin system offers a wide panel of known and yet unknown targets. Some already exist, such as an inhibitor of E1 activity or, more specifically, inhibitor of MDM2 ligase activity or of p53 recognition[37]. Considering that each tumor type is characterized by a specific signature of mutations patterns, genomic alterations, or altered phosphorylations, they may also be characterized by specific alterations in the ubiquitin machinery. From this point of view, pancreatic cancer should have a quite singular signature that would participate in this excessively aggressive phenotype. Therefore, depicting the complete scheme of alterations touching the ubiquitylation/deubiquitylation system in pancreatic cancer cells is surely one promising approach in which to dig in order to find, finally, an efficient drug to treat this disease that has resisted treatments and scientists for too long.
